# Development of the Neonatal Intestinal Barrier, Microbiome, and Susceptibility to NEC

**DOI:** 10.3390/microorganisms11051247

**Published:** 2023-05-09

**Authors:** Alena Golubkova, Catherine J. Hunter

**Affiliations:** Division of Pediatric Surgery, Department of Surgery, University of Oklahoma Health Sciences Center, Oklahoma City, OK 73104, USA

**Keywords:** intestinal barrier, permeability, intestinal epithelial layer, physical barrier, immune response, lamina propria, intraepithelial lymphocytes, mucus layer, microbiome, biotics

## Abstract

The function of the intestinal barrier is partially dependent on host maturity and the colonization patterns of the microbiome to which it is exposed. Premature birth and stressors of neonatal intensive care unit (NICU)-related support (e.g., antibiotics, steroids, etc.) can alter the host internal environment resulting in changes in the intestinal barrier. Pathogenic microbial proliferation and breach of the immature intestinal barrier are proposed to be crucial steps in the development of neonatal diseases such as necrotizing enterocolitis. This article will review the current literature on the intestinal barrier in the neonatal gut, the consequences of microbiome development for this defense system, and how prematurity can influence neonatal susceptibility to gastrointestinal infection.

## 1. Introduction

Microbial dysbiosis and an immature intestinal barrier are established factors that are associated with an increased susceptibility of newborns to inflammatory gastrointestinal diseases, such as necrotizing enterocolitis (NEC). This may explain, in part, disease severity in the very preterm infant, given that they have both an underdeveloped intestinal immune system and increased barrier leakiness. The intestinal barrier is a complex interplay of physical and immune defense layers primed against pathogen invasion. Studies on intestinal barrier function mainly focus on the intestinal epithelial cell (IEC) layer and its physical qualities controlling permeability, the protective mucus layer overlying the intestinal epithelium, and the immediate components of the immune system in the underlying lamina propria, as well as the interspersed intraepithelial lymphocytes within the IEC layer. These barrier components associate with the muscularis mucosae and comprise the internal lining of the small bowel known as mucosa. 

A full-term infant is first exposed to maternal microflora during delivery. Preterm infants, born via cesarean section, lack this initial step, and differences in commensal microbial colonization are described [1,2]. Furthermore, additional stressors of preterm care, including prolonged NICU stay, separation from the mother, respiratory support, antibiotic use, formula feeding, anti-acid administration, prolonged periods on total parenteral nutrition (TPN), and periods of fasting, all contribute to the development of microbial dysbiosis [2,3,4,5]. Likewise, a newborn’s digestive system is exposed to external stimuli with ingested nutrition and, based on its maturity at the time, may either exhibit functional integrity or predisposition to infection. It is established that a newborn infant receives optimal nutrition and immune support from their mother through breast milk [6,7]. Bioactive factors in breast milk are also key to promoting development of the intestinal barrier [7]. 

**Figure 1:**
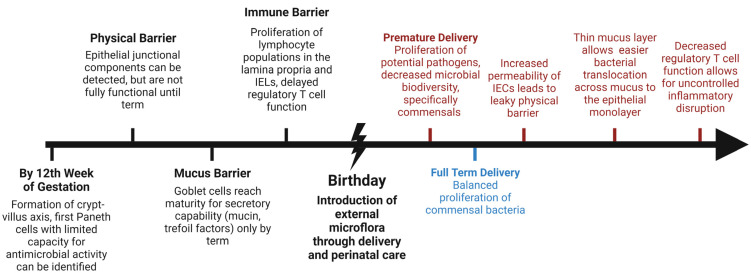
Timeline of development of the intestinal barrier and potential entry points for pathogen invasion due to prematurity of a barrier layer (red). Premature birth (red) is associated with microbial dysbiosis, while full-term delivery (blue) has been found to favor balanced proliferation of a healthy microbiome. (IEL intestinal epithelial lymphocytes, IEC intestinal epithelial cells). Created with BioRender.com.

Functional maturity of the barrier is dictated by its selective permeability, readiness for a controlled immune and inflammatory response, and the ability to efficiently repair itself if injury occurs. The neonatal gut microbiome develops through interactions of the new host gut with microflora that are introduced through feeding (Figure 1). Microbial interactions are crucial to the normal maturation and development of the neonatal gut. However, microbial interactions have the potential to be pathogenic or beneficial. For example, abnormal colonization with a propensity for *Enterobacteriaceae* sp. early after birth is associated with the development of NEC, whereas probiotic supplementation or balanced proliferation of commensal bacteria (i.e., *Bifidobacterium* sp.) are associated with a healthy microbiome proliferation. The objective of this review article is to summarize the current theories and research on the dynamic, physical, and immune defense layers that comprise the intestinal barrier, and how immaturity of this system leads to disease in newborns. Furthermore, strategies that are aimed at enhancing the intestinal barrier to prevent or treat gastrointestinal disease in the newborn will be discussed, including augmentation of the previously mentioned defense layers as well as supplementation with biotics in order to promote a healthy microbiome.

## 2. Development and Composition of the Intestinal Barrier

Maturation of the intestinal lining occurs on a continuum starting in utero, and remains ongoing into the postnatal period with exposure and adaptation of the intestinal lumen to the outside world [1]. The intestinal epithelium originates from the endoderm, and at around the 5th week of gestation proliferates and occludes the lumen of the gut, only to recanalize through apoptosis after 7–9 weeks [8]. The crypt–villus axis begins to form villi after the 9th week of gestation, followed by crypt formation at 10–12 weeks [1,8]. While villi substantially increase the absorptive surface of the intestine, crypts are key for maintenance and cell turnover of the intestinal epithelial layer. The average lifespan of a differentiated epithelial cell (except for Paneth cells whose lifespan is 30 days) is up to 5 days [9].

Stem cells from the crypt bases, also known as crypt base columnar cells (CBC) (identified by expression of Lgr5 receptors), proliferate and differentiate into the five cell types of the epithelium [10,11]. The five major cell types that make up the intestinal epithelium are enterocytes, goblet cells, Paneth cells, enteroendocrine cells, and tuft cells [10,12,13]. Enterocytes mainly play an absorptive role in digestion and are the most abundant cell type. Goblet cells are responsible for production and secretion of the mucus that forms the first chemical and dynamic defense layer of the intestinal barrier, the mucus barrier. Goblet cells comprise 4–12% of the cells of the intestinal epithelium [12]. Paneth cells remain in the crypts and protect stem cells by participating in signaling cascades that regulate cellular proliferation/differentiation and keep the surrounding microbiome in check. Enteroendocrine cells secrete gastrointestinal and peptide hormones that regulate physiologic functions such as digestive enzyme secretion and motility [12]. Tuft cells were originally identified as being chemosensory in nature and present in the intestinal epithelium at around 20–22 weeks of gestation, and are similarly expressed in the respiratory epithelium and biliary system [14]. They are also key modulators of the immune response against parasitic infections through production of IL-25, which induces proliferation and activation of type 2 innate T cells [13,15]. Their role in the pathophysiology of inflammatory gastrointestinal disease such as NEC is undefined, although it has been proposed that their ability to produce prostaglandins may allow them to modulate inflammatory responses [13,16]. 

**Figure 2:**
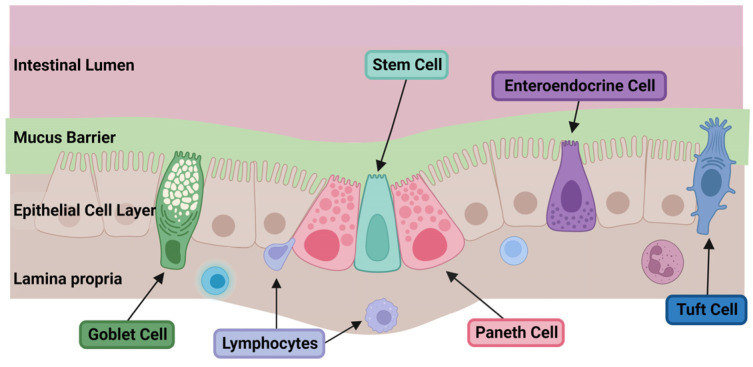
Simplified depiction of the layers and cells of the intestinal barrier. The intestinal barrier is composed of chemical, physical, and immune defense layers. From the apical side facing the intestinal lumen, the first is a chemical barrier of mucus which covers the underlying well-appositioned epithelial cells which create the physical barrier of the intestine. The epithelial monolayer is composed of the following main cell types: absorptive enterocytes, mucin-secreting goblet cells, Paneth cells, stem cells, tuft cells, and enteroendocrine cells. The most basal layer underlying the epithelial cells is the lamina propria, which is the immune barrier composed of gut-associated lymphocytes. Created with BioRender.com.

The intestinal epithelium, represented by the variety of cell types described above, makes up the second major layer of the intestinal barrier, and largely regulates the physical permeability of the defense system. The lamina propria underlies the intestinal epithelium and represents the third layer of this gut defense, responsible for the immune response to factors and pathogens that cross the mucus and epithelial barriers (Figure 2).

## 3. The Mucus Barrier 

The mucus barrier is the outermost defense layer of the intestinal barrier, and is produced and maintained by goblet cells of the underlying intestinal epithelium. It is a thick layer of mucus composed mainly of high molecular weight, hydrophilic glycoproteins, or mucins that form a dynamic barrier over the intestinal epithelium and prevent its direct contact with intraluminal pathogens [17,18]. This barrier is dynamic, as it is continuously synthesized and its coverage or thickness can be modified in response to interactions with external antigens. Additionally, sugar components and, eventually, the protein backbone of the mucins can be broken down and used as nutrients by commensal and pathogenic bacteria, thus competitively regulating the gut microbiome populations as a nutrition resource [19,20,21,22]. 

Thus far, twenty-one different mucin genes have been identified, with mucin 2 (MUC2) being the major expressed component of mucus [23]. Mucins are organized by whether they are secreted or membrane-bound to the epithelium. Additionally, secreted mucins are defined as either gel-forming or not [24]. MUC2 is a gel-forming, secreted mucin that can bind and inhibit microbial translocation and has been found to be vital for a well-functioning mucus barrier in the intestine and colon [19]. The importance of MUC2 for intestinal defense is confirmed with MUC2 knockout murine models that develop spontaneous colitis [25]. Secreted mucins, such as MUC2, are able to polymerize into large oligomers via a variety of chemical bonds and links [26]. Variable assembly of the mucin network is responsible for the viscoelastic, gel-like barrier properties of mucus [27,28]. Additionally, the viscosity of mucus is regulated not only by the concentration of mucins, but also by other components such as proteins (i.e., trefoil factor, IgA) and DNA content. Lower concentrations of total protein, DNA, and mucin, lead to decreased viscosity and increased permeability of the barrier, and thus potential towards microbial invasion of the underlying epithelium [12].

Immediately after birth, the mucus barrier of the neonatal intestine is immature and its protective function against pathogenic invasion is thought to be lacking. Decreased mucus thickness and quantity is associated with a more permeable mucus layer, and a thinner layer is associated with a heightened potential for bacterial translocation [29]. Rat pups, exposed to *E. coli* on day 2 after birth, show signs of bacteria in the blood within 48 h of exposure. The proposed route of infection is through an area of the small bowel that is yet to develop a full mucus layer. This susceptibility decreases with increasing age up to day 9 after birth. Interestingly, this is associated with maturation of the mucus barrier to full thickness by day 9 in the rat model [29]. Another study compared rat ileum of 5-day- versus 21-day-old pups showing an increase in the amount of mucus present in the older pups [17]. In addition, composition analysis of the mucus showed increased amounts of mucin, DNA, and IgA in the older ileum, which are hypothesized to increase the viscosity of the barrier. Passive diffusion and bacterial transport across the mucus barrier was faster in 5-day-old ileum, confirming that maturity and composition of the mucus barrier may regulate ease of bacterial translocation. Goblet cell density can partly explain the decreased production of mucus early in the postnatal period. This has been demonstrated in a preterm piglet model, where, compared to term pigs, preterm piglets had a lower goblet cell density and thus a decreased ability to secrete mucus [30]. In parallel to this translational data, intestinal specimens of human infants with NEC show significantly decreased expression of MUC2 (as well as another mucin, MUC1) on immunohistochemical analysis [31].

In addition to mucins, goblet cells also produce and secrete trefoil factors into the mucus. Trefoil factors are peptides key to regulating and promoting barrier restitution, and they have been shown to prevent enterocyte apoptosis and are upregulated with cellular proliferation after injury or during active inflammatory disease [32,33,34]. Fecal proteomic analysis of gastrointestinal-barrier-related proteins from preterm and term infants shows a significantly lower abundance of trefoil factor 2 (TFF2), trefoil factor 3 (TFF3), and mucosal immune protein, mucin-5AC (MUC5AC), in preterm infants [18]. MUC5AC is a gel-forming mucin that is thought to interact with another trefoil factor, TFF1, uniquely co-upregulated in the gut affected by inflammatory bowel disease, and likely has a role in clearance of pathogens as well as promotion of barrier repair [35]. Of note, MUC5AC deficiency in the colon leads to increased injury and inflammation in mice subjected to experimental DSS (dextran sulfate sodium)-induced colitis [36]. However, based on the previously mentioned proteomic studies in preterm infants, decreased expression of the above trefoil factors and MUC5AC appears to remain dampened throughout the first 6 weeks of postnatal life in preterm as compared to term infants [18]. This suggests that the preterm intestinal barrier not only has an increased susceptibility to microbial invasion, but also impaired repair pathways to deal with the injury caused by infection.

## 4. The Intestinal Epithelial Barrier

The intestinal epithelial barrier lies immediately below the mucus layer and forms the second defense line of the intestinal barrier. It is a single cell layer of epithelial cells that are aligned side by side, forming the physical border of the mucosa. The side of the epithelium facing the intestinal lumen is known as the apical border, while the basal border is on the opposite side facing the lamina propria. Epithelial cells are attached to their neighboring cells by intercellular proteins known as the apical junctional complex (APC). Composition of the apical junctional complex is critical in the modulation of permeability across the cell border. Three types of intercellular junctional proteins are the tight junction components (i.e., claudins, occludin, junctional adhesion molecules, zonula occludens), adherens junction proteins (i.e., E-cadherin), and desmosomes. These proteins connect to the cytoskeleton to create a support system across the epithelial monolayer [37]. While all three are important for structural integrity of the epithelial border, tight junction proteins are particularly vital for paracellular permeability.

Two routes of paracellular ion and solute transport have been defined across the epithelial monolayer. They are known as the pore and leak pathways [37]. The pore pathway is regulated mainly by claudin expression, while the more selective leak pathway is influenced by occludin, zonula occludens-1 (ZO1), and myosin light chain kinase (MLCK) [38]. Changes in junctional protein expression are linked to changes in permeability and consequently disease-susceptible states known as a leaky gut. Frank injury, inflammation, and necrosis of the intestine erode and disrupt the paracellular epithelial pathways completely, leading to unrestricted movement across this physical barrier. Claudin-2 is an example of a pore-forming tight junction protein whose increased expression is associated with increased paracellular permeability in inflammatory bowel disease. Conversely, occludin is a barrier-forming tight junction protein, whose downregulation is associated with increased permeability in inflammatory bowel disease [37]. Furthermore, pathogenic bacterial interaction with the intestinal epithelial barrier can augment permeability of the epithelial monolayer. Specifically, adherent invasive *Escherichia coli* (AIEC) interaction with junctional proteins is thought to be a major contributor to the pathologic gut permeability seen in inflammatory bowel disease [39]. 

It has been proposed that changes in tight junction protein composition, either from interactions with the newly introduced microbiome versus immaturity of the apical junctional complex between intestinal epithelial cells of preterm infants, lead to increased permeability across the physical barrier of the gut, thus contributing to the leaky gut phenomenon and pathogenesis of NEC. One of the widely accepted NEC induction hypotheses is based on the premise that TLR4 (Toll-like receptor 4) signaling within the intestinal epithelium is required for intestinal barrier breakdown and disease development [40]. Experimental NEC models utilize a Gram-negative bacterial ligand, lipopolysaccharide (LPS), to induce NEC. LPS interacts with TLR4 receptors on the basal surface of the intestinal epithelium to set off the inflammatory cascade seen in NEC. Therefore, ready paracellular transport of LPS across the epithelial barrier to the basal side is required in order for the model to work [2]. This is further demonstrated by the changes seen in expression of pore-forming claudin-2 and barrier-tightening claudin-3 and claudin-4 after NEC induction in Caco-2 cells (human epithelial cells isolated from the colon with a history of colorectal adenocarcinoma) and formula-fed rat pups exposed to hypoxia and bacterial contamination. Upregulation of claudin-2 and downregulation of the latter two claudins results in increased permeability as assayed by FITC (fluorescein isothiocyanate) [41]. Finally, bacterial pathogens such as *Cronobacter sakazakii* (formerly known as *Enterobacter sakazakii*), tied to specific NEC outbreaks related to formula contamination, have been shown to disrupt the intestinal epithelial barrier directly by the elicited inflammatory response and increased enterocyte apoptosis in an IEC-6 cell (rat intestinal epithelial cells) model as well as rat pup NEC model [42]. 

In addition to providing the physical barrier and regulation of solute/ion permeability, the intestinal epithelial barrier is also home to Paneth cells (PCs). Paneth cells reside within crypts and mainly serve as the protectors of intestinal stem cells by releasing antimicrobial factors, thereby fending of local infection [43]. Paneth cells are typically present by twelve weeks of gestation, but become fully mature several months later into the postnatal period [9]. Their main function is secretory, releasing factors involved in signaling that promote stem cell proliferation and differentiation as well as regulation of the gut microbiome. Factors released by Paneth cells are known as antimicrobial peptides (AMPs), and include the alpha-defensins and lysozyme. These provide innate mucosal immunity to the intestine against potential pathogens. Alpha-defensins have antibacterial and antiviral properties and function by inducing perforation of the bacterial membrane. Lysozyme is abundant in breast milk and functions by triggering hydrolysis and breakdown of bacterial cell walls. It is proposed that production and secretion of these antimicrobial factors is reduced in preterm infants as compared to term, secondary to the immaturity of the Paneth cells. Mucosal immunity is age-dependent, and lysozyme production is decreased with younger age. In a mouse model, lysozyme levels were significantly reduced in newborn mice as compared to adult mice, and did not reach adult levels until around 28 days of life, which correlates with increased susceptibility to infection prior to that [44]. Similar findings of gradual upregulation of lysozyme secretion can be followed in humans over the first year of life [44]. An earlier human study looking at lysozyme production showed that tissues from NEC-affected patients had very few to no lysozyme-positive Paneth cells on immunohistochemistry as compared to healthy newborns [45].

Dysfunction of Paneth cells offers an alternative hypothesis to the pathogenesis of NEC, although it has mostly been presented in murine models. The classically accepted theory is that bacterial invasion of the immature intestinal epithelium leads to inflammation, necrosis, and disruption of the intestinal barrier; a “top down” approach. The alternative to this is represented as a “bottom up” approach. McElroy and colleagues have postulated that destruction or dysfunction of Paneth cells in the crypts is what leads to the consequent inflammation, increased propensity for microbial invasion, and bowel necrosis [46]. Their lab has developed a murine model (wild-type CD1 mice) in which selective destruction of Paneth cells with dithizone (zinc chelator) injections induced an inflammatory reaction that led to NEC-like pathology, especially with exposure to a potential bacterial pathogen, such as *Klebsiella pneumoniae* [47]. Interestingly, this NEC model does not require TLR4 activation, an established requirement in several other animal models of NEC [48]. Because Paneth cell maturation lags in early development, it is reasonable to propose that the limited function of these cells makes the preterm neonatal gut more vulnerable to infection. In contrast, Heida et al. quantified Paneth cell populations in human ileal tissue from infants between the gestational age of 9 and 40 weeks. They identified that a rise in Paneth cells is most obvious at term gestational age in infants, while the number of immune-competent Paneth cells becomes more pronounced after 29 weeks of gestational age. The group postulated that the rise in PC competency coincides with an increased risk of NEC at 29–33 weeks, which may be the result of an excessive inflammatory response [49]. Further work is required to identify the role of Paneth cell dysfunction versus dysregulation as one of the causes of NEC.

## 5. The Immune Barrier: Lamina Propria, Intraepithelial Lymphocytes (IEL), and Peyer’s Patches

The immune layer of the intestinal barrier lies immediately below the intestinal epithelial monolayer. The mucosal immune system is divided into inductive and effector tissue structures (i.e., Peyer’s patches and the lamina propria, respectively) [50]. The lamina propria is a technical term for the basement membrane that houses the microvasculature, neural network, and key immune system players (effector site of the immune barrier) that identify and destroy pathogens that have breached both the mucus and the intestinal epithelial barriers. T lymphocytes of the lamina propria are first noted after around 12–14 weeks gestation and develop populations comparable to the full-term gut by around 19–27 weeks of gestation [51]. In comparison, the first B lymphocytes are seen around 14 weeks, some of which undergo IgA class-switch after birth. IgA+ plasma cells only reach adult population levels by around 2 years of age. IgA concentrations in the serum reach adult levels by the second decade [51]. Thus, human breast milk is a valuable source of immunoglobulins for preterm and newborn full-term infants [52]. Theories regarding the role of the immune barrier in preterm susceptibility to NEC have mainly focused on dysfunction of the anti-inflammatory properties of lymphocytes, specifically regulatory T cells and intraepithelial lymphocytes. 

The function of regulatory T cells (Tregs) in the pathogenesis of NEC is another pathway that is potentially disrupted, leading to unregulated inflammation and cell death after microbial invasion. Tregs express the transcription factor FOXP3 and are critical for suppressing an overwhelming hyperinflammatory response. Small intestinal tissue samples from infants affected by NEC demonstrate significantly lower Treg populations [53].

The effector mucosal immune system also offers another lymphocyte population that is interspersed among the epithelial barrier: the intraepithelial lymphocytes (IEL). Intraepithelial lymphocytes expand their population after birth [51]. Populations of IELs are significantly decreased in NEC-affected human ileum [54]. A select group of IELs, with the receptor γδ, are preferentially decreased in NEC tissue in humans and mice, with consequently more severe injury phenotype, higher inflammatory TNFα expression, and decreased levels of IL17, a cytokine vital for balancing the inflammatory response [54]. 

While the lamina propria is the main site of action for effector immune cells, Peyer’s patches serve as compartments were antigens are introduced to naïve immune cells (inductive tissue), so lymphocytes learn to recognize pathogens and be primed to respond to potential infection. Peyer’s patches are organized islands of immune cells that are mostly found in the jejunum and ileum, and rarely in the duodenum. Antigen recognition and introduction at the Peyer’s patches mainly occurs with the assistance of microfold (or M) cells, another specialized intestinal epithelial cell that overlies Peyer’s patches in the small intestine [55]. M cells facilitate uptake of antigens into the Peyer’s patch, in order to present the foreign, unwanted particle to the underlying antigen-presenting cells, which further sets off priming and stimulation of lymphocytes for memory and effector activity, teaching the immune defense layer to prevent further breach of the intestinal barrier [56]. An early study on the distribution and characteristics of Peyer’s patches in the human small intestine showed a gradual increase in the number of patches seen with increasing gestational age (24 weeks to 14 years of age) [57]. Furthermore, maturation of the lymphoid tissue is only really prompted postnatally, with stimulation of the patches with introduction of external environmental microbial stimuli (i.e., delivery route, nutrition, NICU-related therapies). Although not confirmed in the literature, it is proposed that immaturity of the inductive tissue of the mucosal immune defense contributes to increased susceptibility of a neonate to gastrointestinal infection [58].

## 6. Microbiome of the Maturing Gut

Although far from being completely understood, maturation of the intestinal barrier is not only dependent on intrinsic programing of the barrier, but is also closely influenced by the developing microbiome after birth. Introduction to commensal and pathogenic bacteria primes the immune cells for future response and establishes a microbial population balance. Exposure to different modes of delivery (cesarean vs. vaginal) primes neonates for different microbial colonization patterns [59]. A healthy, full-term infant’s gut is preferentially dominated by *Bifidobacteria* [60]. Colonization of term gut is initially dominated by *Streptococcus*, *Staphylococcus*, *E. coli*, *Lactobacillus*, and *Enterobacter* species, which are then replaced with anaerobes such as *Bifidobacteria* once the former consumes available oxygen [5]. Another recent study proposed that the intestinal environment is anaerobic at birth and does not require oxygen consumption by facultative anaerobes in order to facilitate consequent microbiome changes with proliferation of obligate anaerobes [61]. In comparison, premature infants have been shown to have decreased and delayed colonization of the gut by commensal microbes [62]. An analysis following changes in the establishment of gut microbiota in preterm (30–35 weeks gestational age at birth) versus full-term infants (38–41 weeks gestational age at birth) showed that preterm infants tend to have higher levels of facultative anaerobes and reduced levels of beneficial anaerobes such as *Bifidobacteria* and *Bacteroides* spp. [63]. 

Additionally, artificial support measures that are frequently required for the care of the preterm infant, such as ventilation and positive airway pressure respiratory support, further disrupt the natural environment that supports proliferation of healthy anaerobic microflora. Instead, the increased aerobic environment supports growth of facultative anaerobes that are pathogenic and invasive in the face of an immature intestinal barrier, such as the *Klebsiella* and *Enterococcus* species [18]. Prolonged fasting and total parenteral nutrition are other examples of therapeutic strategies that can alter the typical microbiome development of a premature infant. There is significant loss of microbial diversity in infants maintained on TPN for nutrition. Additionally, this loss of diversity preferentially targets beneficial bacteria such as *Bifidobacteria* [64]. Empiric antibiotic administration is also a common management strategy in low birth weight, premature infants that has the potential to be an instigator for NEC development by modulating the gut microflora growth. A systematic review and meta-analysis recently demonstrated that prolonged initial (first dose within the first three postnatal days) empiric antibiotic therapy (≥5 days of treatment) is associated with increased NEC risk [65]. Another study looking at permeability markers as a measure of intestinal barrier maturity showed a potential trend between increased barrier permeability and prolonged antibiotic use (>4 days) [66]. However, this study was unable to distinguish whether this effect is a result of delayed feeding patterns, antibiotic use, or a synergistic consequence of both on the developing microbiome. Finally, Chabaan et al. have demonstrated in their murine model that early systemic antibiotic administration has the potential to impair intestinal maturation (decreased cell proliferation, impaired crypt–villi development, and decreased goblet and Paneth cell numbers) and led to increased intestinal permeability and inflammatory baseline. Mouse pups who were exposed to antibiotics (ampicillin/gentamicin) and then an oral bacteria challenge (*Klebsiella pneumoniae*) were more likely to develop NEC-like intestinal injury, likely due to a weakened mucus barrier [67]. 

Although NEC is a result of microbial dysbiosis in the gut, there has been no single pathogen identified as the prominent culprit for disease development. Instead, the gut of NEC-susceptible preterm infants is more commonly associated with lower microbial diversity than that of a full-term infant [68]. When looking at the intestinal flora of infants affected by NEC, bacterial proliferation is also overwhelmed by a variety of Gram-negative bacteria [2,69]. Of interest, a study utilizing 16s rRNA gene sequencing to characterize the developing microbiome of preterm infants (<33 weeks) from birth, to NEC diagnosis, and then 1–2 weeks after treatment, showed a higher microbial abundance at birth and increased populations of “waterborne bacteria” in NEC-susceptible infants compared to preterm infants who did not develop the disease [70]. The authors of this study suggested that preterm infants susceptible to NEC are more easily affected by the bacterial diversity their gut is exposed to from the surrounding environment, and careful attention should be paid to contamination that is potentially coming from water sources utilized by the hospitals with neonatal intensive care units. Moreover, even though NEC likely has a polymicrobial pathogenesis, *Cronobacter sakazakii*, a powdered neonatal formula contaminant, has been linked to several NEC outbreaks in the United States [71]. *C. sakazakii* is a Gram-negative facultative anaerobe, resistant to desiccation. Hunter et al. have shown that this microbe is able to induce intestinal epithelial apoptosis and increase intracellular inflammatory cytokine production (interleukin-6, IL-6) in a rat pup NEC model and in the IEC-6 cell line, thus leading to epithelial barrier damage and disruption [42]. A recent study has further determined that *C. sakazakii* is able to induce NEC inflammation and cellular death through interaction with the TLR4 signaling pathway, activation of intracellular NF-κβ, and upregulation downstream of the NLRP3 inflammasome that triggers pyroptosis and inflammatory disruption of the intestinal barrier [72].

## 7. Breast Milk and Biotics

Microbial components found in infant nutrition have a direct interaction with the maturing intestine. Two major preventative strategies aimed at strengthening the intestinal barrier and promoting a healthy microbiome in the infant have been areas of active investigation. The first is breastfeeding and the second is biotic (pre- and pro-biotic) supplementation of infant feeds. 

### 7.1. Breast Milk

The value of breastmilk as a source of optimal nutrition and immune support for a neonate is clearly established [7]. For example, human milk oligosaccharides (hMOs) are the third most abundant component of breast milk and have been shown to confer multiple benefits to the developing neonatal gut intestinal barrier and microbiome [73]. There are five known monomers that make up hMOs (D-glucose, D-galactose, N-acetylglucosamine, L-fucose, and sialic acid) [74]. HMOs serve as a nutritional supplement to promote healthy growth of commensal bacteria. They also resemble the sugars found in the mucus barrier of the intestine. Certain pathogens utilize the sugars found in mucus as anchors to bind and cross the mucus barrier. The binding of these microbes to hMOs prevents adhesion and translocation across the mucus barrier, thus serving as “decoy receptors” and an additional defense mechanism for the intestinal barrier [74]. In such a way, intestinal inflammation seen with *Campylobacter jejuni* invasion is attenuated with hMO supplementation, as demonstrated in infected human cell lines and four-week-old mice who have been pretreated with antibiotics and then exposed to the pathogen [75]. HMOs also have a beneficial effect on intestinal epithelium, particularly in the face of TLR4-triggered inflammation. TLR4 upregulation and IL-8 expression were suppressed in a murine NEC model when pups were supplemented with hMOS. Additionally, epithelial cell proliferation was rescued and improved despite LPS exposure in an enteroid NEC model [76]. Thus, hMOs are able to provide nutrition for commensal bacteria, prevent pathogenic invasion, and promote intestinal barrier repair. 

Breast milk provides not only key nutrients for the developing baby and healthy commensal bacteria proliferation, but also immune factors that support the immune system that is not yet optimally functional. First, breast milk is a source of IgA (Immunoglobulin A) produced by B cells in maternal mammary glands that have originated from the mother’s gut-associated lymphoid tissue, and are thus primed against intestinal microbes the mother has been exposed to [77]. A recent study by Gopalakrishna et al. demonstrated that maternal IgA confers critical protection against NEC. They showed that breastfed mice pups were more susceptible to NEC when mothers were IgA-deficient, despite being breastfed. Moreover, they have shown that onset of NEC is dominated by IgA-unbound *Enterobacteriaceae*, suggesting that maternal IgA is important for controlling proliferation of pathogenic bacteria prior to disease induction [78].

Two other breast milk components have recently been closely studied for their potential to strengthen the mucus barrier defense: lysozyme (LYS), which is secreted by Paneth cells, and docosahexaenoic acid (DHA), a polyunsaturated fatty acid. Both are abundant in breastmilk but rarely found in commercial formula. LYS and DHA supplementation of rat pups with experimentally induced NEC (early cesarean delivery, hypoxia exposure, formula feeding, and bacterial colonization) demonstrated an improved mucus barrier with decreased permeability and bacterial translocation across the layer [17]. Additionally, breastfeeding and breastmilk serve as a direct seeding source of gut microflora from the mother to the baby and thus have a key role in neonatal microbiome development when breastfeeding is possible [79]. Studies on breastmilk microbiome composition show that milk is a source of *Staphylococcus*, *Streptococcus*, and *Pseudomonas* spp., which likely originate from skin contact, but also demonstrate that translocation of bacteria from a mother’s gastrointestinal tract into the breastmilk occurs. Furthermore, the health of a mother (i.e., obesity, perinatal infections, mode of delivery, obstetric complications) can dictate which bacterial strains dominate and are potentially passed to the infant [80,81,82,83]. Moreover, maternal dietary habits, such as a high-fat diet, are known to be associated with changes to the neonatal microbiome, specifically decreased representation of *Bacteroides* spp. [84]. A systematic review attempted to identify maternal dietary patterns and association with microbiome changes, confirming that diets rich in fat are associated with reduced neonatal microbial diversity. In contrast, maternal fiber intake is associated with increased biodiversity in the neonatal microbiome [85]. Thus, counseling on dietary augmentation for the mother during pregnancy has the potential to influence beneficial neonatal microbiome development.

### 7.2. Biotics 

The second strategy for prevention of neonatal gastrointestinal disease involves pre- or probiotic supplementation. Prebiotics are usually nutritional supplements that enhance growth of beneficial bacteria, while probiotics are actual live cultures of beneficial bacteria. A combination of both is known as a synbiotic [86]. For instance, the previously mentioned components of breastmilk, hMOs, are considered a prebiotic or growth factor for commensal *Bifidobacteria* (“bifidus factor”) [87]. Safety and efficacy of oligosaccharide supplementation to preterm infants has been investigated through a systematic review which concluded that supplementation is well tolerated by the infants, improves stool quality, and increases growth of beneficial *Bifidobacteria* but it is not significantly associated with a lower incidence of NEC [88]. 

In a similar fashion, research has focused on probiotic administration to preterm infants for introduction and support of healthy microbial growth in the gut. A majority of studies investigating the benefits of probiotics on infant health utilize multistrain combinations of probiotics versus single strain. Multistrain combinations are potentially more efficacious. *Lactobacillus* and *Bifidobacterium* spp. are the most commonly included strains [89]. A systematic review has concluded that routine use of probiotics is associated with significantly reduced Stage ≥2 NEC incidence and all-cause mortality in premature infants (<37 weeks gestational age), and just NEC incidence in extremely low birth weight infants (<1000 g), without the similar benefit to mortality seen in the former group [89]. In contrast, another meta-analysis of 18 randomized controlled studies showed the benefit of probiotic supplementation was evident in decreased incidence of sepsis, but not NEC [90]. Probiotic supplementation of formula-fed infants may also ameliorate the negative immune-related effects associated with a lack of breastfeeding [91]. Additionally, early supplementation with *B. infantis* of infants exclusively fed breast milk results in persistent colonization with beneficial *Bifidobacteria* at one year after birth, suggestive of the long-term benefit of gut microbial homeostasis with early routine probiotic supplementation [92].

It is proposed that proliferation of commensal bacteria in the gut can regulate the potential hyperinflammatory response to a pathogenic breach in the intestinal barrier [93]. Underwood et al. demonstrated that in a rat pup NEC model (disease induction with formula feeds, hypoxia, and cold stress), supplementation with *Bifidobacterium longum* subspecies *infantis* (*B. infantis*) of rat pups on formula reduced experimental NEC incidence and expression of inflammatory cytokines and markers (i.e., IL-6, TNF-a, and iNOS) [93]. Probiotics supplementation also has the potential to downregulate enterocyte apoptosis after NEC injury, thus maintaining epithelial layer integrity [94]. Lin et al. utilized supplementation with the probiotic strain *Lactobacillus rhamnosus GG* (LGG) to show downregulation of apoptosis in IEC-6 cells exposed to proapoptotic staurosporine (STS), as well as in chemically induced apoptosis in the intestine of two-week-old mice [94]. Finally, Blackwood et al. confirmed with their studies in Caco-2 cells and rat pups that supplementation with *Lactobacillus rhamnosus* and *Lactobacillus plantarum* was protective against tight junction-disrupting stimuli. Specifically, improved (lower permeability) trans-epithelial resistance (TER) measurements were demonstrated in treated Caco-2 cells, and reduced intestinal injury was seen in rat pups exposed to *C. sakazakii*. Furthermore, decreased permeability (measured by FITC) was demonstrated in rat pups treated with both the pathogen and probiotics. Interestingly, permeability was not affected in the same way with only probiotic administration, suggesting that the benefit of probiotic administration is only evident when a potential pathogen exists [95]. Thus, probiotics may be valuable in maintaining the physical barrier of the epithelium and preventing a leaky gut in the face of NEC-induction.

Despite the purported benefits of probiotic supplementation, there are several prominent arguments against routine use of probiotics in premature infants [96]. Studies showing probiotic supplementation benefits are limited by the variety of probiotic strains that have been studied, inadequate power, lack of large randomized-control trials, and the variation in chosen infant study populations. Additionally, there is no single, accepted regimen of probiotics, as the supplement is not FDA (Food and Drug Administration)-regulated and varies from batch to batch given its origin as a live bacterial strain. Thus, a standardized, universally safe probiotic regimen is yet to be adopted for regular preventative use in the NICU. Alternatively, maternal instead of neonatal supplementation with pre- and probiotics during pregnancy is another potential route for indirectly modulating the developing baby’s microbiome [97]. Eating a fiber-rich diet has been discussed earlier as a prebiotic to promote microbial diversity in the neonate. The benefit of maternal probiotic supplementation was also demonstrated in a translational murine study where pregnant mouse dams were administered *Lactobacillus acidophilus* and *Bifidobacterium infantis*. Their mouse pups were then challenged with intraperitoneal injections of IL-1β to induce an inflammatory insult. Pups whose mothers took probiotics demonstrated improved gut barrier maturation and decreased levels of inflammatory cytokines, confirming the anti-inflammatory effects of maternal probiotics on fetal gut health [98]. 

## 8. Conclusions

The intestinal barrier is a complex, layered mechanism that functions as a dynamic chemical, physical, and immune defense system against pathogenic microbial invasion. Multiple systems have to develop at variable times during gestation in order for the barrier to confer the full protection it should. Premature birth is a stressor that exposes the intestinal barrier to external stimuli prior to its full functional development. Additionally, care strategies aimed at supporting a preterm infant are disruptive to the natural development of the microbe–gut host environment. Consequently, an immature and unprepared intestinal barrier as well as microbial dysbiosis increase susceptibility of a preterm neonate to gastrointestinal disease. Continued development of strategies aimed at enhancing intestinal barrier maturation and promotion of microbial homeostasis in the gut will likely significantly improve digestive health of the infant and prevent development of infections such as NEC.

## Data Availability

Not applicable.
